# scHSC: enhancing single-cell RNA-seq clustering via hard sample contrastive learning

**DOI:** 10.1093/bib/bbaf485

**Published:** 2025-09-22

**Authors:** Sheng Fang, Xiaokang Yu, Xinyi Xu, Jingxiao Zhang, Xiangjie Li

**Affiliations:** Center for Applied Statistics, School of Statistics, Renmin University of China, Beijing 100872, China; Center for Applied Statistics, School of Statistics, Renmin University of China, Beijing 100872, China; Department of Biostatistics, Epidemiology and Informatics, University of Pennsylvania, Philadelphia, PA 19104, United States; School of Statistics and Mathematics, Central University of Finance and Economics, Beijing 100081, China; Center for Applied Statistics, School of Statistics, Renmin University of China, Beijing 100872, China; National Clinical Research Center for Cardiovascular Diseases, State Key Laboratory of Cardiovascular Disease, Fuwai Hospital, National Center for Cardiovascular Diseases, Chinese Academy of Medical Sciences and Peking Union Medical College, Beijing 100037, China

**Keywords:** single-cell RNA-seq, deep clustering, hard sample mining, contrastive learning

## Abstract

Single-cell RNA sequencing (scRNA-seq) provides high-throughput information about the genome-wide gene expression levels at the single-cell resolution, bringing a precise understanding on the transcriptome of individual cells. Unfortunately, the rapidly growing scRNA-seq data and the prevalence of dropout events pose substantial challenges for clustering and cell type annotation. Here, we propose a deep learning method, scHSC, that employs hard sample mining through contrastive learning for clustering scRNA-seq data. Focusing on hard samples, this approach simultaneously integrates gene expression and topological structure information between cells to improve clustering accuracy. By adjusting the weights of hard positive and hard negative samples during the iterative training process, scHSC employs an adaptive weighting strategy to integrate contrastive learning with a ZINB model for single-cell clustering tasks. Extensive experiments on 18 single-cell RNA-seq real datasets demonstrate that scHSC exhibits significant superiority in clustering performance compared to existing deep learning-based clustering methods. scHSC is implemented in Python based on the PyTorch framework. The source code and datasets are available via https://github.com/fangs25/scHSC.

## Introduction

Single-cell RNA sequencing (scRNA-seq) technology allows researchers to measure gene expression levels at the resolution of a single cell, which is crucial for characterizing cell types [1], studying developmental biology [2], uncovering complex diseases [3], and inferring cell trajectories [4]. Now identifying cell type accurately has become a critical step in scRNA-seq analysis [5]. Clustering has been demonstrated as one of the most effective methods for cell type annotation because it identifies cell types in an unbiased manner [6]. In earlier studies, traditional clustering methods such as K-means [7], hierarchical clustering [8], and density-based clustering [9] have been applied to tackle clustering problems.

However, clustering analysis remains a highly challenging task due to the inherent characteristics of scRNA-seq data. These data are characterized by both high heterogeneity across cell populations and extreme sparsity, with a substantial number of zero elements resulting from technical limitations and biological factors [10, 11].

Thus, developing efficient computational methods to fully leverage the potential of scRNA-seq data is urgently needed.

To address these limitations, researchers have developed various methods for clustering scRNA-seq data. For instance, CIDR [12] is a fast PCA-based algorithm that leverages dissimilarity matrices for imputation and clustering. SC3 [13] proposed a consensus clustering framework specifically for single-cell RNA-seq data, which employs PCA and Laplacian transformations to reduce the dimensionality of genes. SIMLR [14] uses multi-kernel learning to find a more robust distance metric, aiming to address the prevalent dropout events. However, these computational methods often tend to provide locally optimal results on scRNA-seq data, as they fail to account for the extreme sparsity caused by gene expression levels. Moreover, most of them rely on constructing complete graph Laplacian matrices, which incurs significant computational and storage costs. Although the problem remains challenging, recent works have advanced solutions via graph learning, multimodal integration, and deep clustering [15–17].

In recent years, deep embedded clustering methods have been successfully developed to model high-dimensional and sparse scRNA-seq data like DESC [18], scDeepcluster [19], scDCC [20], and scziDesk [21]. These methods iteratively refine clusters by learning highly reliable assignments using auxiliary target distributions, achieving improved clustering results. Meanwhile, other deep learning models such as DCA [22] have focused on robust representation learning by denoising raw expression data, which benefits downstream tasks including clustering and visualization. More recent approach, such as scMAE [23], have introduced masked autoencoding frameworks for clustering, where a proportion of input gene expression values are randomly masked and then reconstructed by the model.

However, these deep embedded clustering methods often overlook structural information propagation and node relationships. Recently, emerging graph neural networks have demonstrated their ability to naturally capture graph structural information through neighbor information propagation [24], as demonstrated by scTAG [25], scMGCA [26], and scGAE [27] in single-cell analysis. Graph embedding clustering typically combines deep autoencoders and graph clustering algorithms to learn compact latent representations, enabling the exploration of both rich content and structural information [28]. However, those methods suffer from poor scalability due to full-graph construction, over-smoothing in noisy data, and reliance on static neighborhood graphs that don’t adapt during training—limiting their performance on large or complex datasets.

In addition, contrastive learning has also been successfully applied to clustering problems in scRNA-seq, such as scDCCA [29], graphSCC [24], scDSC [30], and contrastive-sc [31]. Although these methods have achieved promising clustering results, they do not consider hard positive and hard negative samples in deep metric learning, which limits the improvement of clustering performance. HSAN [32] proposed a strategy in graph clustering that adjusts the weights of hard positive and hard negative samples, but it is only suitable for simple graph data and cannot be applied to complex scRNA-seq data.

Single-cell RNA-seq data presents major challenges for clustering due to its high sparsity, technical noise, and cell-type heterogeneity. While recent deep learning methods have made progress, many still suffer from over-smoothing, poor scalability, or static neighborhood assumptions that limit adaptability during training. To address these limitations, we propose scHSC, a **H**ard-**S**ample aware **C**ontrastive clustering framework tailored for scRNA-seq data. scHSC adopts a sampling-based training strategy and dynamically refines neighborhood structures using high-confidence pseudo-labels, offering improved scalability and robustness in clustering performance. Our method jointly integrates gene expression profiles and graph topology, and dynamically emphasizes hard positive and hard negative pairs during training to refine the embedding space. The design of scHSC directly addresses the challenges of sparsity and heterogeneity in single-cell data by focusing on hard positive and hard negative samples. In sparse datasets, truly similar cells may appear dissimilar due to dropouts (creating hard positives), while heterogeneous populations may yield deceptively similar profiles among distinct cell types (creating hard negatives). By assigning more attention to these confusing but informative samples during training, scHSC improves the discriminability and robustness of learned embeddings. We conducted experimental evaluations on 18 real scRNA-seq datasets, and the results demonstrate that scHSC exhibits significantly superior clustering performance compared to other methods.

## Materials and methods

### Framework of scHSC

scHSC employs a contrastive learning strategy to iteratively identify hard sample pairs based on inter-sample similarity relationships. By dynamically adjusting sample weights, it enables a more comprehensive capture of cellular relationships, which facilitates downstream analyses.

**Figure 1:**
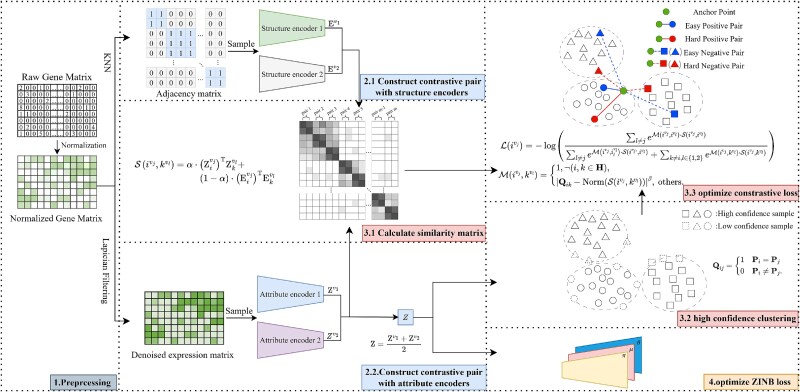
**Framework of scHSC**. scHSC primarily comprises three components: a contrastive learning module (Steps 2.1 and 2.2), a hard sample weighting module (Steps 3.1–3.3), and a ZINB-based autoencoder (Step 4). The preprocessed gene expression matrix is first fed into the attribute encoders, while the KNN graph is input to the structure encoders, resulting in two attribute embeddings and two structure embeddings, respectively. These four embeddings are then jointly used in two ways: (1) they are fed into the hard sample weighting module to adaptively assign sample weights based on the similarity matrix, and (2) they serve as input to the ZINB-based autoencoder to effectively model the high sparsity inherent in scRNA-seq data. More implementation details can be found in the Materials and Methods section.

As illustrated in Fig. 1, scHSC primarily consists of three modules: an augmentation-free contrastive learning module, a Zero-Inflated Negative Binomial (ZINB)-based autoencoder and a hard sample weighting module. The adjacency matrix and expression matrix, both derived from the preprocessed gene count matrix, are fed into two structure encoders and two attribute encoders, respectively, generating two structure embeddings and two attribute embeddings.

These four embeddings are integrated to produce a fused similarity matrix for subsequent sample weighting. Specifically, the fused attribute embedding serves dual purposes: it is passed through a ZINB decoder to preserve the high-dimensional sparse characteristics of scRNA-seq data, and it supports high-confidence clustering to generate pseudo-labels for the cells.

Utilizing the fused similarity matrix and pseudo-labels of the cells, scHSC dynamically adjusts the weights of all sample pairs when calculating the contrastive loss—assigning higher weights to hard sample pairs and lower weights to easy sample pairs.

### Data preprocessing

We applied standard preprocessing procedures to the raw count matrix using Scanpy. Genes and cells with fewer than one count were first removed using *sc.pp.filter_genes* and *sc.pp.filter_cells* with *min_counts=1*. We then normalized the total counts per cell using *sc.pp.normalize_total* with default settings, which scales each cell’s total count to the median of total counts across all cells prior to normalization. Following normalization, we applied a logarithmic transformation using *sc.pp.log1p* to stabilize variance. We identified the top $p$ highly variable genes using Scanpy’s *seurat* method. Unless otherwise specified, we selected $p=2000$ genes. The resulting expression matrix was finally standardized to zero mean and unit variance using *sc.pp.scale*. This preprocessing yielded a normalized matrix $X \in \mathbb{R}^{N \times p}$, where $N$ is the number of cells and $p$ is the number of selected genes.

### Topological structure and Laplacian filter

To capture the intrinsic topological structure between cells before model training and other potential information loss, we construct an undirect graph $\mathcal{G} = (\mathcal{V,E})$ based on matrix $X$ using the K-nearest neighbor (KNN) method, where $\mathcal{V}$ represents the set of $N$ nodes (cells) and $\mathcal{E}$ denotes the set of edges between cells. We can simply use the adjacency matrix $A \in \mathbb{R}^{N \times N}$ to characterize this graph structure, where each element $A_{ij}$ indicates the connectivity between cells $i$ and $j$. Specifically, $A_{ij} = 1$ if cell $j$ is within the KNNs of cell $i$, and $A_{ij} = 0$ otherwise.

Graph Laplacian filtering has been widely used in image data denoising and attributed graph embedding learning [33, 34]. In the context of high-dimensional, sparse scRNA-seq data, we found this strategy helps to smooth gene expression values between neighboring cells, ensuring that cells with similar expression patterns have more comparable features after filtering. Specifically, given an adjacency matrix $A$, we can compute the degree matrix $D = \text{diag}\{d_{1},d_{2},\ldots ,d_{N}\} \in \mathbb{R}^{N\times N}$, where $d_{i} = \sum _{v_{j} \in \mathcal{V}} A_{ij}$ is the degree of node $v_{i}$. The graph Laplacian matrix $L$ of $A$ is defined as $L = D - A$. With the renormalization trick $\tilde{A} = I + A$, we have the symmetric normalized graph Laplacian:


\begin{align*} \tilde{L}_{\text{sym}} = \tilde{D}^{-\frac{1}{2}} \tilde{L} \tilde{D}^{-\frac{1}{2}}, \end{align*}

where $\tilde{D}$ and $\tilde{L}$ are degree matrix and Laplacian matrix corresponding to $\tilde{A}$.

Subsequently, applying the Generalized Laplacian Smoothing Filter to smooth the expression matrix [34], the filtered matrix is presented by


\begin{align*} X_{\text{filtered}} = \left(I - \tilde{L}_{\text{sym}}\right) X = \tilde{D}^{-\frac{1}{2}} \tilde{A} \tilde{D}^{-\frac{1}{2}} X. \end{align*}

### Fused contrastive learning module

Contrastive learning is a self-supervised learning approach that aims to learn representations by comparing similar and dissimilar pairs of data points. The fundamental principle is to maximize the similarity between positive pairs and minimize the similarity between negative pairs. The contrastive strategy offers the ability to learn meaningful representations without the need for labeled data and it can enhance robustness to noise and capture fine-grained structures within the data. Recent research has demonstrated its effectiveness in representation learning, achieving promising results in both image data and single-cell data analysis [31, 35].

Instead of masking some data as zeros for data augmentation [29], we input the filtered data $X_{\text{filtered}}$ into two independent attribute encoders $f_{Z_{1}}, f_{Z_{2}}$ to generate two distinct $d$-dimensional attribute embeddings $Z^{v_{1}}, Z^{v_{2}}$ of the same cell, which are then used to construct sample pairs. The use of two independent encoders ensures that $Z^{v_{1}}$ and $Z^{v_{2}}$ capture complementary yet distinct aspects of the cell’s attributes. Similarly, we input the adjacency matrix into two independent structure encoders $f_{E_{1}}, f_{E_{2}}$ to obtain two distinct structure embeddings $E^{v_{1}}, E^{v_{2}}$. This dual-encoder strategy enhances the robustness of the learned representations by introducing diversity in both attribute and structure spaces. The construction and functionality of positive and negative sample pairs are further detailed and mathematically formulated in Equation (1). It is worth noting that, for the sake of model generalization and computational efficiency, we sample $m$ cells from the overall dataset ($A,X_{\text{filtered}}$) without replacement at each iteration, resulting in two submatrices: $A_{\text{sub}} \in \mathbb{R}^{m\times N}, X_{\text{sub}} \in \mathbb{R}^{m\times p}$. Thus, we have the overall formula below:


\begin{align*} \left.\begin{array}{l@{}} Z^{v_{1}} = f_{Z_{1}}(X_{\text{sub}}), Z^{v_{2}} = f_{Z_{2}}(X_{\text{sub}}) \\ E^{v_{1}} = f_{E_{1}}(A_{\text{sub}}), E^{v_{2}} = f_{E_{2}}(A_{\text{sub}}) \\ \end{array}\right\} \in \mathbb{R}^{m\times d}. \end{align*}

Using these four $d$-dimensional embeddings, we compute a fused similarity matrix $\mathcal{S}$ to facilitate subsequent hard sample contrastive learning:


\begin{align*} \mathcal{S} =& \begin{pmatrix} \mathcal{S}_{11} \quad \mathcal{S}_{12} \\ \mathcal{S}_{21} \quad \mathcal{S}_{22} \end{pmatrix} = \alpha \cdot \begin{pmatrix} Z^{v_{1}}(Z^{v_{1}})^\top \quad Z^{v_{1}}(Z^{v_{2}})^\top \\ Z^{v_{2}}(Z^{v_{1}})^\top \quad Z^{v_{2}}(Z^{v_{2}})^\top \end{pmatrix} \\&+ (1-\alpha) \cdot \begin{pmatrix} E^{v_{1}}(E^{v_{1}})^\top \quad E^{v_{1}}(E^{v_{2}})^\top \\ E^{v_{2}}(E^{v_{1}})^\top \quad E^{v_{2}}(E^{v_{2}})^\top \end{pmatrix} \in \mathbb{R}^{2m\times 2m}, \end{align*}

where $\alpha $ is the weight of fusing attribute information and structure information. $\mathcal{S}_{11}$ and $\mathcal{S}_{22}$ represent the similarity between sample embeddings of the same version, while $\mathcal{S}_{12} = \mathcal{S}_{21}^\top $ indicates the similarity between sample embeddings of different versions.

That is, the similarity between embeddings of samples across versions is given by the formula below:


\begin{align*} \mathcal{S}(i^{v_{k}}, j^{v_{l}}) = \alpha \cdot (Z_{i}^{v_{k}})^\top Z_{j}^{v_{l}} + (1-\alpha) \cdot (E_{i}^{v_{k}})^\top E_{j}^{v_{l}}, \end{align*}

where $Z_{i}^{v_{k}}, E_{i}^{v_{k}}$ represents the attribute embedding and structure embedding of the $i$-th cell in the $k$-th version, $i,j \in \{1,2,\ldots ,m\}$ and $k,l\in \{1,2\}$.

### Hard sample weighting module

In addition to being used for calculating the fused similarity matrix, these two attribute embeddings will also be utilized in the ZINB autoencoder module in the next section and high-confidence clustering in this section. The high-confidence clustering not only selects high-confidence samples for reweighting, but also generates pseudo-labels that, combined with sample similarity, allow for a precise identification of hard and easy sample pairs. Hard sample pairs consist of samples from the same class but with low similarity, as well as samples from different classes but with high similarity. In contrast, easy sample pairs consist of samples from the same class with high similarity, and samples from different classes with low similarity. This precise distinction enables us to focus on hard samples while leveraging clear supervisory signals for robust and stable training.

Given the fused attribute embedding $\boldsymbol{Z} = \frac{1}{2}(\boldsymbol{Z}^{v_{1}} + \boldsymbol{Z}^{v_{2}}) \in \mathbb{R}^{m\times d}$ and the target number of clusters, we employ leiden or Louvain method to obtain the pseudo-labels $\boldsymbol{P} \in \mathbb{R}^{m}$ for the cells, with the resolution parameter determined via binary search. To select high-confidence samples, we calculate the cluster center for each class by averaging the embeddings of cells within the same class. Next, we compute the Euclidean distance between each cell’s embedding and the cluster center of its assigned class. The top $\tau \%$ of samples with the shortest distances are selected to form the high-confidence sample set $\boldsymbol{H} \in \mathbb{R}^{m\tau }$, where $\tau $ is set to 90 by default. The pseudo cluster indicator matrix $\boldsymbol{Q} \in \mathbb{R}^{m\times m}$ is determined by the pseudo-labels $\boldsymbol{P}$ as follows:


\begin{align*} \boldsymbol{Q}_{ij} = \begin{cases} 1 \qquad P_{i} = P_{j}, \\ 0 \qquad P_{i} \neq P_{j}. \end{cases} \end{align*}

Given the gene expression profiles of $ m $ cells as input to two attribute encoders, we obtain $ 2m $ attribute embeddings. For a specific embedding $ i^{v_{k}} $ of cell $ i $ in version $ v_{k} $, it forms a positive pair with its counterpart embedding $ i^{v_{l}} $ from the other version $ v_{l} $ of the same cell. In contrast, $ i^{v_{k}} $ forms negative pairs with the remaining $ 2(m-1) $ embeddings derived from all other cells across both versions. Therefore, the widely used InfoNCE loss function in contrastive learning for a positive pair $(i^{v_{k}}, i^{v_{l}})$ is formulated as follows [31, 35, 36]:


(1) \begin{gather*} \mathcal{L}_{\text{InfoNCE}} (i^{v_{k}}) = -\log \frac{e^{\boldsymbol{s}\left(Z_{i}^{v_{k}}, Z_{i}^{v_{l}}\right)}}{e^{\boldsymbol{s}\left(Z_{i}^{v_{k}}, Z_{i}^{v_{l}}\right)} + \sum\limits_{j \neq i, l\in\{1,2\}} e^{\boldsymbol{s}\left(Z_{i}^{v_{k}}, Z_{j}^{v_{l}}\right)}}, \end{gather*}

where $\boldsymbol{s}(\boldsymbol{u},\boldsymbol{v}) = \frac{\boldsymbol{u}^\top \boldsymbol{v}}{\Vert \boldsymbol{u} \Vert \Vert \boldsymbol{v} \Vert }$ is the dot product between $\ell _{2}$ normalized $\boldsymbol{u}$ and $\boldsymbol{v}$ (i.e. cosine similarity). InfoNCE aims to maximize the similarity between positive pairs while minimizing the similarity with negative pairs, effectively learning meaningful representations.

However, this strategy treats all sample pairs with equal weight during training, failing to fully exploit the informative value of hard sample pairs, potentially limiting the representation capability of the algorithm. For example, in clustering tasks, easy samples are often well-separated without requiring much attention, whereas hard samples are more challenging to distinguish and should be prioritized during training. Thus, similar to the HSAN method, scHSC employs a weighting adjustment function $ \mathcal M $ to dynamically adjust the weights of sample pairs throughout the training process [32, 37].

Based on the fused similarity matrix $\mathcal{S}$, pseudo cluster indicator matrix $\boldsymbol{Q}$ and high-confidence sample set $\boldsymbol{H}$, we define the weighting adjustment function $ \mathcal M $ as follows:


(2) \begin{align*} \mathcal{M} (i^{v_{k}}, j^{v_{l}}) = \begin{cases} |\boldsymbol{Q}_{ij} - \text{Norm}(\mathcal{S}(i^{v_{k}}, j^{v_{l}}))|^\beta, &\, i,j \in \boldsymbol{H}, \\ 1, &\, \text{others}, \end{cases}\end{align*}

where $\text{Norm}(\cdot )$ denotes the min-max normalization function, which ensures that the sample similarity lies within the range of $[0,1]$. $\beta $ is the focusing coefficient, where a larger $\beta $ indicates a greater adjustment to the sample weights, meaning more focus is placed on hard sample pairs while less attention is given to easy sample pairs. Finally, we extend the classic InfoNCE loss defined by the Equation (1) to a contrastive loss that focuses on hard sample pairs:


(3) \begin{gather*} \mathcal{L} (i^{v_{k}}) = -\log \frac{e^{\mathcal{S}(i^{v_{k}}, i^{v_{l}}) \cdot \mathcal{M}(i^{v_{k}}, i^{v_{l}})}} {e^{\mathcal{S}(i^{v_{k}}, i^{v_{l}})\cdot\mathcal{M}(i^{v_{k}}, i^{v_{l}})} + \sum_{j \neq i, l\in\{1,2\}} e^{\mathcal{S}(i^{v_{k}}, j^{v_{l}})\cdot\mathcal{M}(i^{v_{k}}, j^{v_{l}}) } }. \end{gather*}

By extending the cosine similarity in Equation (1) to $\mathcal{S}(i^{v_{k}}, i^{v_{l}}) \cdot \mathcal{M}(i^{v_{k}}, i^{v_{l}})$ in Equation (3), we can, on one hand, use the fused similarity to obtain similarity measurements for both structure and attributes, and on the other hand, use the weighting adjustment function to focus more on the information from hard samples while neglecting easy samples. This allows us to fully leverage the power of contrastive learning, efficiently obtaining meaningful representations of high-dimensional sparse single-cell transcriptomic data.

The total hard sample contrastive loss is:


(4) \begin{gather*} \mathcal L_{\text{Hs}} = \frac{1}{2m} \sum_{k=1}^{2} \sum_{i=1}^{m} \mathcal L(i^{v_{k}}). \end{gather*}

### ZINB autoencoder module

The ZINB-autoencoder is designed for scRNA-seq data to address the sparsity and overdispersion of gene expression [22]. It combines a zero-inflated model for handling zeros and a negative binomial distribution to model overdispersed counts, improving robustness and accuracy (ACC) in downstream analyses. To obtain low-dimensional embeddings while preserving data distribution and biological variation, we use the ZINB autoencoder to reconstruct the original count data.

ZINB distribution is a mixture of a negative distribution and a delta distribution, whose probability mass function is given by:


(5) \begin{gather*} \text{NB} (x;\mu,\theta) = \frac{\Gamma(x+\theta)}{\Gamma(x)\Gamma(\theta)} \left( \frac{\theta}{\theta+\mu} \right)^\theta \left( \frac{\mu}{\theta+\mu} \right)^{x}, \notag \\ \text{ZINB} (x;\pi, \mu,\theta) = \pi \delta_{0}(x) + (1-\pi)\text{NB}(x;\mu,\theta), \end{gather*}

where $\mu , \theta $ is mean and dispersion parameter of the negative binomial distribution, and $\pi $ is dropout rate.

Following the structure of DCA [22], we utilizes three separate decoders to estimate three parameters of the ZINB distribution ($\pi , \theta , \mu $) for each gene. The original raw count of each gene is then compared with the ZINB distribution defined by these parameters to calculate a gene-specific loss. Notably, the output dimensions of each decoder match the input dimensions of the encoder, corresponding to the number of genes. Given the fused attribute embedding $\boldsymbol{Z} = \frac{1}{2}(\boldsymbol{Z}^{v_{1}} + \boldsymbol{Z}^{v_{2}})$ mentioned before, the ZINB decoder can be formulated as follows:


\begin{align*} \boldsymbol{\Pi} = \text{sigmoid} (\boldsymbol{Z} \boldsymbol{W}_{\boldsymbol \pi}),\\ \boldsymbol{M} = \exp (\boldsymbol{Z} \boldsymbol{W}_{\boldsymbol \mu}),\\ \boldsymbol{\Theta} = \exp (\boldsymbol{Z} \boldsymbol{W}_{\boldsymbol \theta}), \end{align*}

where $\boldsymbol{W}_{\boldsymbol \pi }, \boldsymbol{W}_{\boldsymbol \mu }, \boldsymbol{W}_{\boldsymbol \theta }$ denote the weights of decoders corresponding to $\pi , \mu , \theta $,respectively, and the matrices $\boldsymbol{\Pi }, \boldsymbol{M}, \boldsymbol{\Theta }$ represent the activated outputs of these decoders. After normalizing $\boldsymbol{M}$ by the library size factor introduced in DCA [22], we naturally take the negative log-likelihood of ZINB distribution as reconstruction loss, which is given by


(6) \begin{align*} \mathcal{L}_{\text{ZINB}} = -\sum_{i=1}^{m} \sum_{j=1}^{p} \log \left[ \text{ZINB}\left(x_{ij}^{\text{raw}};\pi_{ij}, \mu_{ij}, \theta_{ij}\right)\right],\end{align*}

where $x_{ij}^{\text{raw}},\pi _{ij}, \mu _{ij}, \theta _{ij}$ is the raw count, dropout rate estimate, mean estimate and dispersion estimate of gene $j$ in cell $i$, respectively.

Finally, the total loss of scHSC is obtained through Equations (4) and (6), and is given by:


(7) \begin{align*} \mathcal L = \mathcal{L}_{\text{Hs}} + \omega \cdot \mathcal{L}_{\text{ZINB}},\end{align*}

where $\omega $ serves as a balancing coefficient to scale the two loss values to the same magnitude. In the default setting, $\omega $ is dynamically updated throughout training to account for varying loss scales [38, 39]. However, it can also be manually fixed by the user to suit particular experimental requirements. Specifically, Supplementary Table S3 presents clustering performance across four datasets using fixed weights of 1, 5, and 10, as well as the default dynamic weighting strategy in scHSC. Results show that across all datasets, the adaptive weighting strategy achieves consistently high performance, often outperforming or closely matching the best fixed-weight setting.

After training, all cells are forwarded through the network to obtain their attribute embeddings. A KNN graph is then constructed based on these embeddings, and the Louvain algorithm is applied to identify clusters.

## Results

### Datasets and evaluation metrics

In this work, we test scHSC on 18 real scRNA-seq datasets and compared its performance with eight other popular neural network-based or baseline clustering algorithms. The 18 datasets used in the experiment were derived from various organs, including the brain, kidney, and pancreas, as well as from different sequencing platforms, such as 10$\times $, Drop-seq, inDrop, and Smart-seq2. The true cell types of the datasets were provided by the authors, which are typically considered to have high reliability. Detailed information about the datasets is provided in the Supplementary Table S1.

We employed three clustering evaluation metrics: normalized mutual information (NMI), adjusted Rand index (ARI), and ACC to assess the performance of our method and the other eight comparison methods. Higher values of these metrics indicate better clustering performance. Considering that ground-truth class labels are not always available in practice, we additionally introduce two unsupervised, non-parametric metrics—Compactness and Separation—to evaluate clustering quality without relying on reference labels.

Detailed definition of these metrics, please refer to Supplementary Materials.

### Clustering performance comparison with other methods

The clustering methods used for comparison include scTAG [25], scziDesk [21], scDeepCluster [40], DESC [18], DCA [22], scGAE[27], and scMAE [23]. We also add the widely used pipeline of PCA followed by Louvain clustering, which serves as a fundamental benchmark in many single-cell analyses. Most of these methods are based on deep neural networks, particularly autoencoder architectures. Notably, both scTAG and scGAE also incorporate graph-based structure information between cells. All methods were run with their default parameters and the true number of clusters was specified. Furthermore, all experiments were accelerated using an NVIDIA RTX TITAN X GPU with 12 GB of CUDA memory, thereby enhancing computational efficiency.

**Figure 2:**
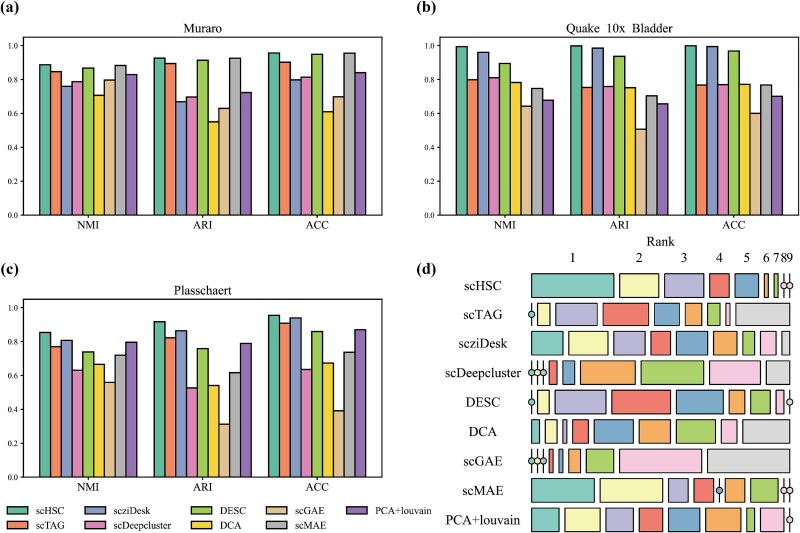
**Clustering performance of scHSC**. (a–c) Comparison of clustering performance (ARI, NMI, ACC) of scHSC and eight competing methods across three representative scRNA-seq datasets; (d) Comprehensive comparison across 18 scRNA-seq datasets using ARI, NMI, and ACC metrics: mosaic plot illustrating method rankings across 54 evaluation indicators with methods as rows and rank levels as columns.

Figure 2a–c presents the NMI, ARI, and ACC results of each method across three representative datasets. Meanwhile, Fig. 2d summarizes the rankings of each method across all 18 datasets and three metrics (NMI, ARI, and ACC) using a mosaic plot. In this plot, each row corresponds to one of the nine methods, and each column represents a ranking position, with the size of the blocks indicating how frequently a given method attains each rank. scHSC consistently ranks in the top three on nearly all datasets and achieves first or second place in the majority of them, highlighting its superior clustering ACC and robustness. For detailed comparison results across all datasets, please refer to the Supplementary Figs S1 and S2, Table S4 and S5. A metric value of zero for a method on a specific dataset in the figure indicates that the method exceeded our server’s CUDA memory capacity during execution, such as scTAG in Bach and Plasschaert datasets.

The selected comparison algorithms exhibit superior performance across our extensive validation datasets. For instance, the NMI scores of scHSC, scMAE, scziDesk, and DESC all exceed 0.90 on the Pollen dataset, while their ARI scores surpass 0.95 on the Quake Smart-seq2 Limb Muscle dataset, indicating near-perfect clustering performance. However, these methods do not maintain consistently strong performance across all datasets. For example, scDeepCluster performs significantly worse than scHSC, scTAG, scziDesk, and DESC on datasets such as Quake Smart-seq2 Diaphragm, Klein, and Plasschaert, while DCA underperforms on datasets like Adam and Muraro. scMAE, scziDesk and PCA+Louvain are the top-performing methods aside from scHSC, achieving the best results on the Macosko, Romanov, and Quake 10$\times $ Limb Muscle datasets, respectively. However, each of these methods also performs noticeably worse than others on certain datasets, such as Quake 10$\times $ Bladder and Adam. In other words, scHSC consistently performs competitively across this wide range of validation datasets, even demonstrating significant improvements on several datasets such as Quake Smart-seq2 Diaphragm and Plasschaert, further emphasizing its outstanding performance and superior robustness. Moreover, as shown in Supplementary Tables S4 and S5, methods such as DCA, scDeepCluster, and scTAG exhibit strong Compactness but relatively weaker Separation, suggesting potential over-clustering tendencies. In contrast, scMAE achieves high Separation but low Compactness, indicating dispersed intra-cluster structure. PCA + Louvain performs poorly on both metrics. Notably, scHSC, DESC, and scziDesk demonstrate balanced performance, with scHSC achieving the best trade-off between intra-class compactness and inter-class separation.

### Visulization result of scHSC

**Figure 3:**
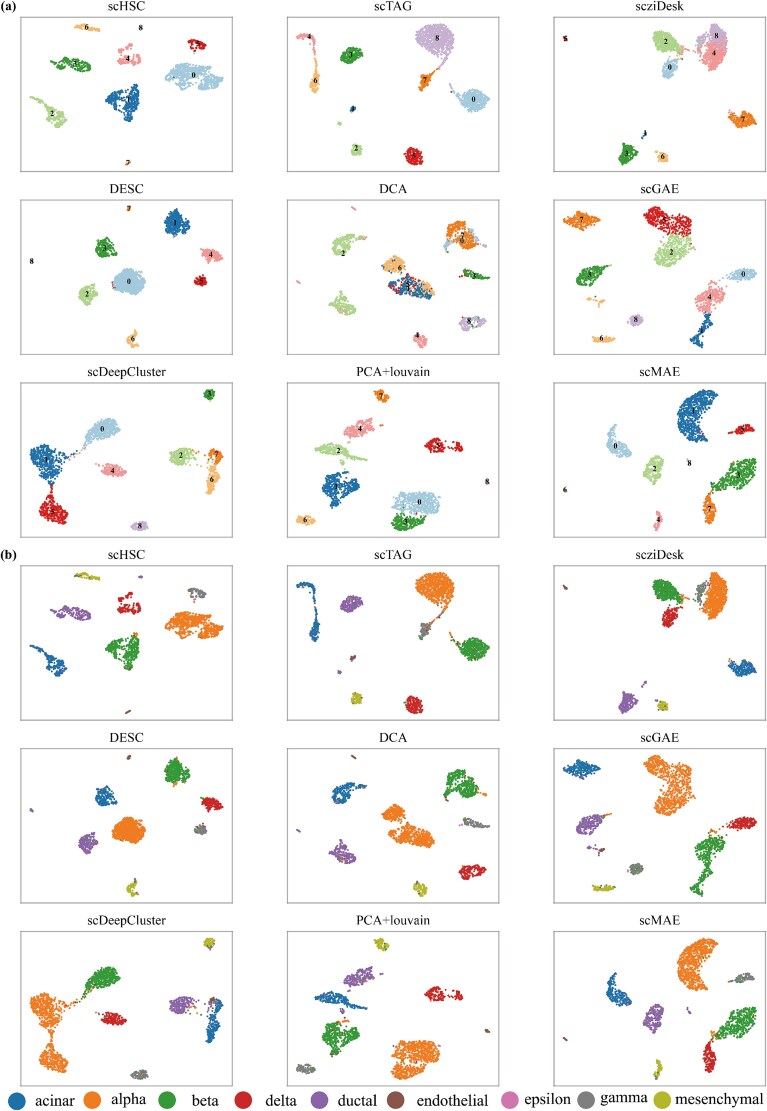
**UMAP visualization on Muraro Dataset**. (a) The 2D UMAP visualizations of the low-dimensional embeddings and the clustering results in the Muraro dataset generated by scHSC and other methods. Each data point in the UMAP plot represents a single cell, with colors indicating the distinct clusters identified. (b) The same as (a) except for colors indicating the distinct cell types in ground truth.

To validate the ability of scHSC to capture true biological variability in high-dimensional sparse sequencing data and demonstrate its clustering performance, we used UMAP to project the low-dimensional embeddings obtained from the bottleneck layer into a two-dimensional space. Figure 3 presents the 2D UMAP visualization of scHSC on the Muraro dataset, where each point represents a cell, and the color indicates the identified clusters in Fig. 3a. The results of the other methods, along with the true cell types, are also plotted in the Fig. 3b.

The Muraro dataset is a human pancreatic dataset that includes nine cell types: alpha, beta, ductal, acinar, delta, gamma, mesenchymal, endothelial, and epsilon. Among these, the endothelial and epsilon cell types are represented by only 21 and 3 cells, respectively. With the exception of the epsilon cell type, which contains only 3 cells, the 2D UMAP visualization of the low-dimensional embeddings generated by scHSC clearly separates the remaining cells into eight distinct subgroups. Notably, it also demonstrates excellent performance in correctly identifying the endothelial cell type, which consists of only 21 cells. scTAG subdivided acinar cells into two distinct groups, while PCA+Louvain, scziDesk and scGAE split alpha cells into two clusters. In addition, DCA and scDeepCluster both performed poorly on identifying acinar and alpha cells. This suggest that, although these methods can partially separate cell populations, they do not fully capture the inherent similarities among cells, resulting in limited performance in distinguishing true cell types.

Overall, scHSC and scMAE demonstrate the best performance on the Muraro dataset. Unlike scTAG and scziDesk, which struggled with certain cell types, scHSC effectively separates cell populations, even in cases with imbalanced or low-abundance cell types. We evaluated the robustness of scHSC against cluster number mis-specification using the Muraro dataset. When varying the number of clusters from 8 to 10, scHSC maintained stable assignments for the majority of cells (Supplementary Fig. S4), demonstrating resilience to slight inaccuracies in cluster number estimation. In addition, results from Supplementary Table S2 show that scHSC is also robust to early-stage pseudo-label noise and network initialization. Controlled experiments confirm that scHSC delivers consistent performance across different initialization strategies and random seeds.We also validated the biological relevance of scHSC by performing marker gene analysis on the Quake 10$\times $ Bladder dataset. As shown in Supplementary Fig. S3, the embeddings and clusters produced by scHSC revealed clear cell-type-specific markers that align well with established biological annotations. These results highlight the method’s robustness and adaptability in single-cell clustering applications.

### Sensitivity and scalability analysis

To evaluate the clustering stability and hyperparameter robustness of scHSC, we performed a robustness analysis on four critical parameters: $\tau $, $k$, $hvg$, and $batch size$. The value of $\tau $ serves as a threshold to select high-confidence pseudo-labeled samples, which are subsequently used to guide the weighting of samples in the hard sample weighting module. The parameter $k$ represents the number of nearest neighbors used in the KNN algorithm to construct the cell graph, which determines the extent of local neighborhood information considered for each cell. The number of $hvg$ (highly variable genes) selected during preprocessing governs the scope of the features included in the model, directly influencing its ability to capture relevant biological variance. Finally, $batch size$ defines the number of cells sampled in each iteration during model training, affecting both the computational efficiency and the model’s capacity for generalization by determining how many samples are processed per update.

**Figure 4:**
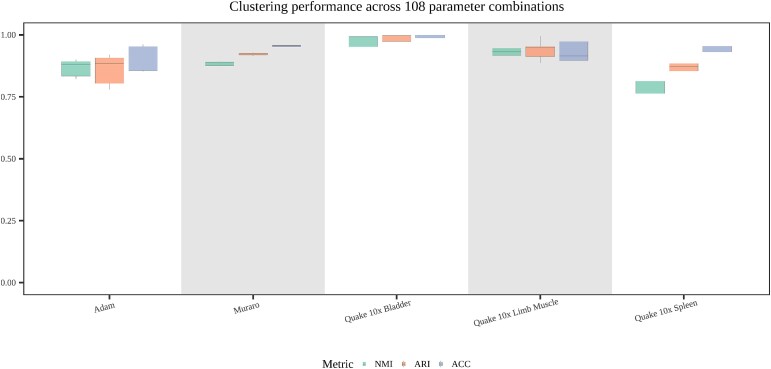
**Sensitivity of clustering performance across 108 parameter combinations for five datasets**. The analysis varies three key hyperparameters: $\tau $ (threshold to select high-confidence samples, 0.85, 0.9, and 0.95), k (number of nearest neighbors, 15, 18, and 20), hvg (number of highly variable genes, 1000, 2000, and 3000), and batch size (200, 500, 1000, and 2000). Each dataset was evaluated using 108 distinct configurations of these parameters ($3 \times 3 \times 3 \times 4 = 108$).

For the robustness analysis, we systematically varied these three key parameters by selecting 3, 3, 3, and 4 distinct values, respectively. Specifically,$\tau $ was set to {0.85, 0.9, 0.95, }, $k$ was set to {15, 18, 20}, $hvg$ to {1000, 2000, 3000}, and $batch size$ to {200, 500, 1000, 2000}. Each dataset was evaluated across all possible combinations of these parameter values, yielding a total of 108 distinct configurations ($3 \times 3 \times 3 \times 4 = 108$). The resulting clustering ACC for each dataset, based on these 108 parameter combinations, is presented in the Fig. 4. As shown in the figure, the performance across the 108 different parameter combinations demonstrates strong consistency and stability for all datasets. The standard deviation of clustering ACC across most datasets is approximately 0.05, indicating that the clustering results remain highly consistent despite variations in hyperparameter settings. Additionally, the ACC levels are consistently high, closely matching those observed with the default parameters. These findings underscore the robustness of scHSC with respect to variations in key hyperparameters, suggesting that the method’s performance is relatively insensitive to these parameters and that it maintains its effectiveness across a wide range of settings.

**Figure 5:**
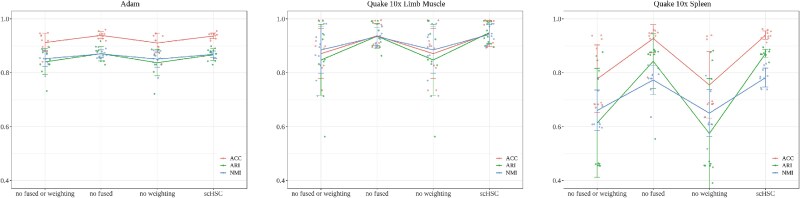
**Ablation study results illustrating the contributions of the fusion similarity matrix and the hard sample weighting module**. The experiments conducted on three representative datasets reveal the distinct roles of the fusion similarity matrix, the hard sample weighting module, and their synergistic combination, respectively. The “no fused or weighting” model neither incorporates fused similarity nor applies hard sample weighting, whereas the “no fused” model exclude the fusion of similarity, and the “no weighting” model omit the use of hard sample weighting. Scatters represent metric scores resulted from runs with different random seeds; values on lines represent the mean over 10 runs; error bars indicate standard deviation.

Furthermore, we conducted ablation studies to evaluate the impact of two crucial components of the model: the fusion similarity matrix (Section 2.4) and the hard sample weighting module (Section 2.5). To assess the contribution of each module, we selected three representative datasets and analyzed the performance with and without these components. The results of these experiments are shown in the Fig. 5. The “no fused or weighting” model neither incorporates fused similarity nor applies hard sample weighting, whereas the “no fused” model exclude the fusion of similarity, and the “no weighting” model omit the use of hard sample weighting. The “no fused” condition corresponds to setting the fusion parameter $\alpha =1$, which disables the integration of structural information and relies solely on the attribute embedding, and the “no weighting” condition means that all sample pairs are treated with equal weight in the contrastive loss. To ensure the robustness and reliability of the ablation results, we conducted each ablation experiment using 10 different random seeds on all three benchmark datasets. We report the mean and standard deviation of the performance metrics across these repeated trials.

As shown in Fig. 5, the hard sample weighting module consistently improves clustering ACC across all three benchmark datasets. Meanwhile, the fused similarity module enhances the model’s robustness by stabilizing performance across trials. To further investigate the impact of the hard sample weighting module under different data conditions, we examined the initial cluster separability of each dataset, quantified by the Separation score at the start of training. Specifically, the Adam dataset exhibited a high initial Separation score of 48, whereas Quake 10$\times $ Limb Muscle and Quake 10$\times $ Spleen showed substantially lower scores of 30 and 20, respectively. When comparing the full model (scHSC) with its ablated variant without the hard sample weighting module (denoted as no weighting), the average clustering ACC dropped modestly by 2% on Adam, but showed more substantial declines of 7% and 19% on Quake 10$\times $ Limb Muscle and Quake 10$\times $ Spleen, respectively. These results indicate that the hard sample weighting mechanism is particularly beneficial for datasets with low initial separability, as it helps emphasize ambiguous or borderline samples that are otherwise difficult to cluster correctly. These results demonstrate that each module plays a important role in refining the model’s ability to capture meaningful biological patterns and improve clustering ACC. The combination of these components is essential for achieving superior performance, particularly when dealing with complex and challenging datasets.

Finally, to assess the computational efficiency of scHSC, we compared its training time and peak memory usage against other methods on the same hardware setup. All experiments were conducted on a server equipped with 32-core Intel(R) Xeon(R) CPU E5-2620 v4 @ 2.10 GHz, a TITAN V GPU with 12 GB memory, and 256 GB system memory.

**Figure 6:**
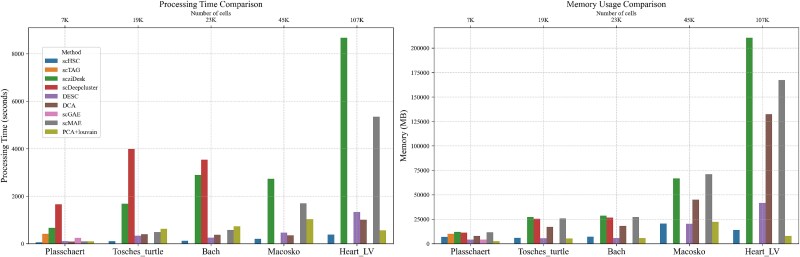
**Runtime and memory comparison**. Runtime and peak memory usage of nine clustering methods across five scRNA-seq datasets of increasing size: Plasschaert (6977 cells), Tosches Turtle (18 664 cells), Bach (23 184 cells), Macosko (44 808 cells), and Heart LV (107 249 cells). The y-axis in the left panel represents runtime (in seconds), while the y-axis in the right panel denotes peak memory usage (in MB).

As shown in Fig. 6, we compared the training time and peak memory usage of nine methods across five datasets of varying sizes: Plasschaert (6977 cells), Tosches Turtle (18 664 cells), Bach (23 184 cells), Macosko (44 808 cells), and Heart LV (107 249 cells). All neural network-based methods were executed with CUDA acceleration, except for scziDesk, which was run on CPU due to implementation constraints (TensorFlow 1.14). In detailed, graph-based methods such as scTAG and scGAE failed to complete clustering on datasets with more than approximately 20 000 cells due to GPU memory overflow. Similarly, scDeepCluster was unable to process datasets exceeding 40 000 cells. Among all evaluated methods, only PCA+Louvain, DESC, DCA, scMAE, and our proposed scHSC successfully completed the clustering task on the 100 000-cell dataset. PCA+Louvain provided a relatively fast solution (about 10 min) with good scalability and ACC, though it requires manual tuning of the resolution parameter, which may compromise reproducibility. DESC and DCA completed the task in around 20 min but required 40–130 GB of memory, while delivering suboptimal clustering performance. scMAE achieved relatively better clustering ACC but needed approximately 90 min and 170 GB of memory. scziDesk was the most resource-intensive method, taking about 140 min and consuming 210 GB of memory. In contrast, our proposed scHSC finished clustering within 7 min, requiring only 14 GB of CPU memory and 3 GB of GPU memory, while achieving strong performance without hyperparameter tuning.

## Discussion

In this study, we propose a hard-sample aware contrastive learning approach to enhance clustering of scRNA-seq data. We evaluated scHSC using a variety of large-scale and diverse scRNA-seq datasets. Compared to established mainstream methods, scHSC achieved superior clustering performance, accurately grouping cells into their respective types. Additionally, scHSC effectively preserved the inherent biological variability present in the data, ensuring that important biological signals were maintained. Furthermore, scHSC demonstrated high stability across key hyperparameters, indicating robustness and reliability in different experimental settings. Moreover, scHSC offers significant advantages in both computational time and memory usage. Thanks to its sampling strategy and adaptive weighting design, scHSC demonstrates superior convergence acceleration and reduced peak memory usage when benchmarked against existing methods, particularly for large-scale datasets. These characteristics make scHSC not only accurate and biologically meaningful, but also practical and scalable for real-world single-cell analysis tasks.

Contrastive learning is a self-supervised machine learning technique designed to learn effective data representations and has emerged as a powerful tool in scRNA-seq analysis for enhancing clustering performance and preserving biological variability. We further incorporate hard-sample contrastive learning into the clustering of single-cell data. By dynamically identifying hard samples and adjusting their weights, we leverage contrastive learning to better differentiate cell populations and enhance the stability of clustering results.

Moreover, we extensively leverage the topological graph relationships among cells. The graph structure was directly input into structural encoders to generate structural embeddings and was also employed for Laplacian smoothing of gene expression data. This methodology ensures the comprehensive preservation of biological variability throughout the training process. Typically, an increased sample size enriches graph-based information by capturing more intricate relationships among cells, thereby enhancing clustering performance. However, managing and utilizing the graph structures of large-scale datasets during training imposes significant demands on memory and computational resources. To mitigate these challenges, we implement a sampling strategy that reduces memory and computational requirements by utilizing only a subset of data and structural information during training. Nonetheless, this approach may constrain the model’s ability to capture more comprehensive global structural information.

Contrastive learning effectively extracts features by leveraging positive and negative sample pairs, demonstrating significant promise in scRNA-seq analysis. In this study, we applied contrastive learning to enhance clustering performance and preserve biological variability across large and diverse datasets. Our results highlight the method’s capability to address the complexities inherent in single-cell data. In future studies, we will explore the applications of contrastive learning in mitigating batch effects and integrating multi-omic data, thereby further improving the robustness and comprehensiveness of single-cell analyses.

**Key Points**

- scHSC effectively integrates gene expression and topological structure information between cells, using them to construct sample pairs for contrastive learning and build fused similarity for hard sample mining.
- scHSC fully leverages the power of contrastive learning by precisely identifying hard samples in the clustering task and dynamically adjusting their training weights, resulting in efficient cellular representations.
- scHSC outperforms state-of-the-art methods in both accuracy and robustness, demonstrating superior clustering performance across diverse scRNA-seq datasets from multiple platforms and organs. It also shows clear advantages in memory efficiency and computational time on large-scale datasets, highlighting its scalability for practical applications.

## Supplementary Material

Supplementary_Materials_bbaf485

## Data Availability

All datasets used in this study are publicly available. The Adam (GSE94333), Bach (GSE106273), Klein (GSE65525), Macosko (GSE63473), Muraro (GSE85241), Plasschaert (GSE102580), and Romanov (GSE74672) datasets can be obtained from the Gene Expression Omnibus (GEO). The Pollen dataset (SRP041736) is available from the NCBI Sequence Read Archive (SRA). The Tabula Muris dataset, which includes profiles generated using SS2 and 10X platforms, is accessible at Tabula Muris website, with seven selected datasets used in this study. The Tosches turtle dataset can be downloaded directly from Tosches Turtle page, and the Young dataset is available from the CELLxGENE database.The Heart LV dataset can be downloaded from https://www.heartcellatlas.org/. All processed datasets are available for download at Googld Drive.

## Code availability

scHSC is implemented in Python based on the PyTorch framework. The code and datasets are available via https://github.com/fangs25/scHSC.
