# Supplementary material for: scHSC: enhancing single-cell RNA-seq clustering via hard sample contrastive learning
Source: Brief Bioinform. 2025 Sep 22;26(5):bbaf485. doi: 10.1093/bib/bbaf485 (PMC12451106; doi:10.1093/bib/bbaf485)
Supplement: Supplementary_Materials_bbaf485 [file supplementary_materials_bbaf485.pdf]

Supplementary Materials for scHSC: Enhancing Single-Cell RNA-Seq Clustering via Hard Sample Contrastive Learning

Sheng Fang, Xiaokang Yu, Xinyi Xu, Jingxiao Zhang, Xiangjie Li

Figure S1: Clustering Performance for all 18 datasets

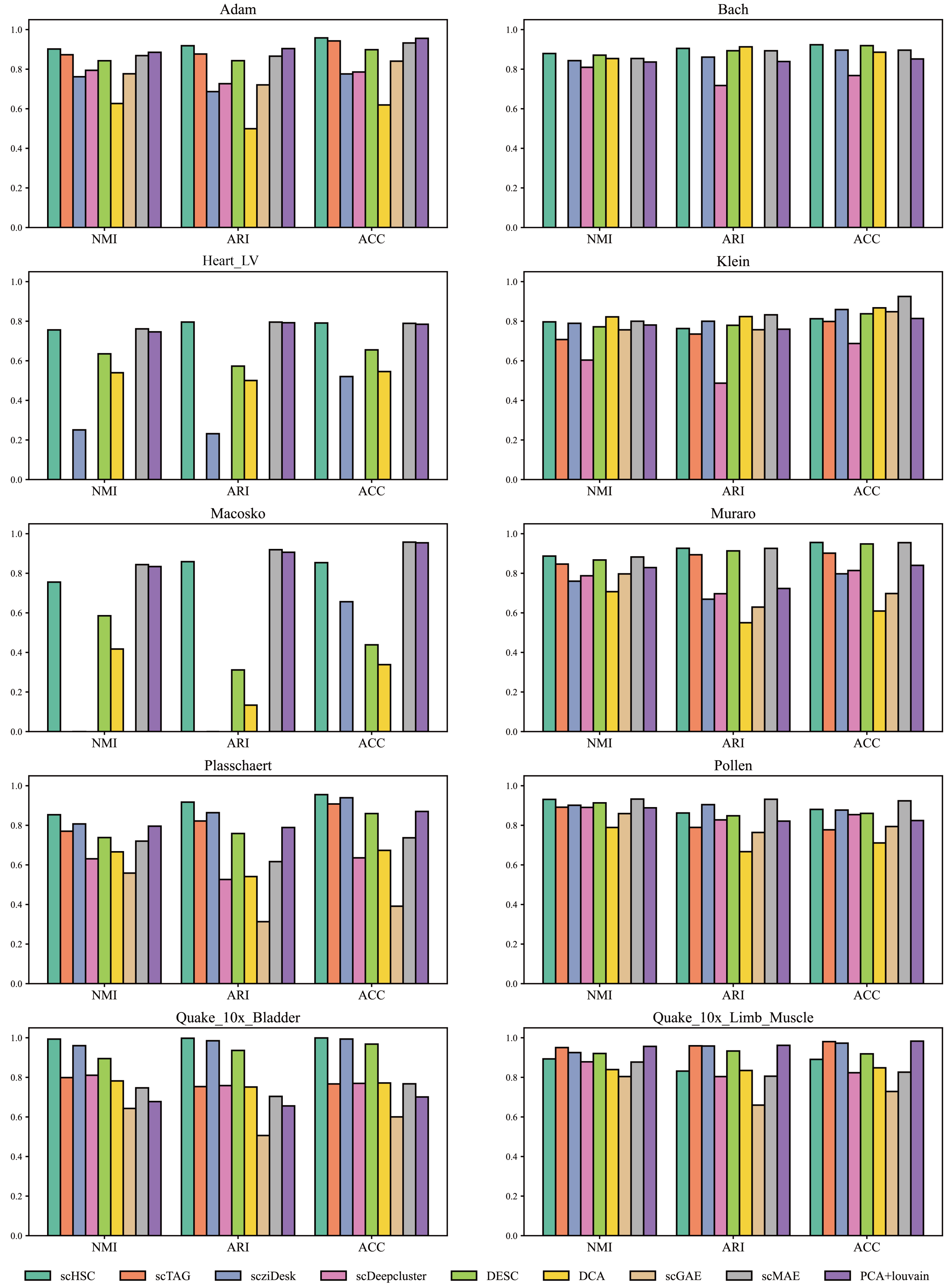

Figure S2: Clustering Performance for all 18 datasets

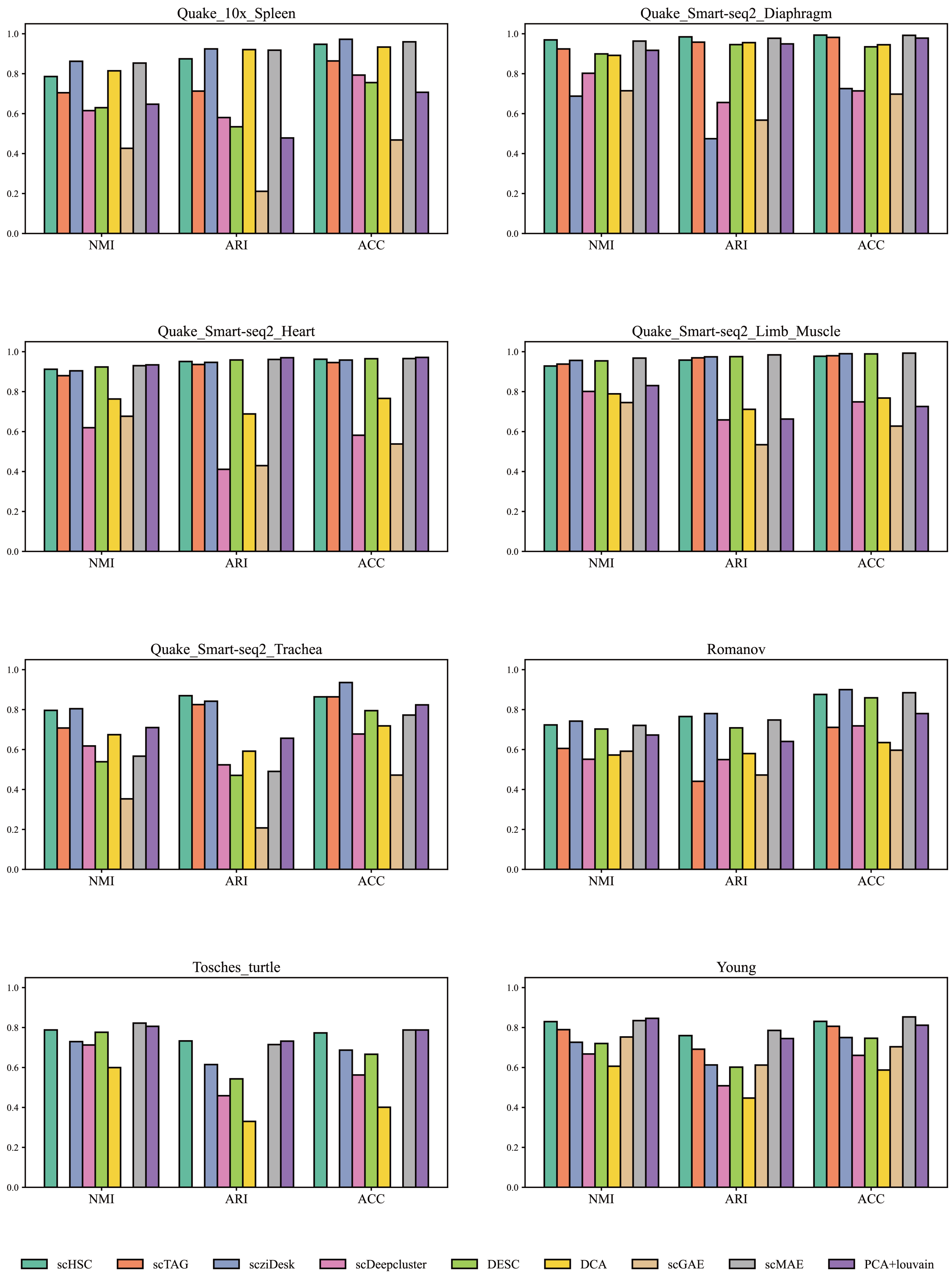

Evaluation Metrics

Given a set of true labels $\mathbf{y} = [y_1, y_2, \ldots, y_n]$ and the corresponding clustering labels $\mathbf{c} = [c_1, c_2, \ldots, c_n]$, we evaluate the clustering performance using three metrics: Normalized Mutual Information (NMI), Adjusted Rand Index (ARI), and Clustering Accuracy (ACC). The non-parametric metric Compactness and Separation are also included.

Normalized Mutual Information (NMI)

The Normalized Mutual Information (NMI) between the true labels $\mathbf{y}$ and the clustering labels $\mathbf{c}$ is defined as:

$$\mathrm{NMI}(\mathbf{y}, \mathbf{c}) = \frac{I(\mathbf{y}, \mathbf{c})}{\sqrt{H(\mathbf{y}) \cdot H(\mathbf{c})}},$$

where the mutual information $I(\mathbf{y}, \mathbf{c})$ is given by:

$$I(\mathbf{y}, \mathbf{c}) = \sum_{i=1}^{n} \sum_{j=1}^{n} p(y_i, c_j) \log \frac{p(y_i, c_j)}{p(y_i) \cdot p(c_j)},$$

and the entropy of the true labels $\mathbf{y}$ and the clustering labels $\mathbf{c}$ are:

$$H(\mathbf{y}) = -\sum_{i=1}^{n} p(y_i) \log p(y_i), \quad H(\mathbf{c}) = -\sum_{j=1}^{n} p(c_j) \log p(c_j),$$

Adjusted Rand Index (ARI)

The Adjusted Rand Index (ARI) is computed as:

$$\mathrm{ARI}(\mathbf{y}, \mathbf{c}) = \frac{\mathrm{RI} - \mathbb{E}[\mathrm{RI}]}{\max(\mathrm{RI}) - \mathbb{E}[\mathrm{RI}]},$$

where the Rand Index (RI) is defined as:

$$\mathrm{RI} = \frac{a + d}{\binom{n}{2}},$$

with a being the number of pairs of samples that are in the same cluster and belong to the same true class, and d being the number of pairs that are in different clusters and belong to different true classes.

Clustering Accuracy (ACC)

The Clustering Accuracy (ACC) is defined as:

$$\mathrm{ACC} = \frac{1}{n} \sum_{i=1}^{n} \mathbf{1}[y_i = \sigma(c_i)],$$

where $\sigma(c_i)$ is the best label mappings from the clustering labels to the true labels using Hungary algorithm, and $\mathbf{1}(\cdot)$ is the indicator function.

Compactness

Compactness measures the intra-cluster dispersion by computing the sum of squared distances between each sample and its assigned cluster center. Given a dataset $X = \{x_1, x_2, \ldots, x_n\} \in \mathbb{R}^d$ and the corresponding clustering labels $\mathbf{c} = [c_1, c_2, \ldots, c_n]$, partitioned into K clusters with centers μ_k, the compactness is defined as

$$\frac{1}{n} \sum_{k=1}^{K} \sum_{x_i \in C_k} ||x_i - \mu_k||^2,$$

where $C_k = \{x_i | c_i = k\}$.

Separation

Separation quantifies the inter-cluster dispersion by measuring the squared distances between all pairs of cluster centers and Separation is defined as

$$\frac{1}{K} \sum_{1 \leq i < j \leq K} ||\mu_i - \mu_j||^2,$$

where K represents the number of clusters and μ_k represents center of cluster k.

Table S1: All 18 datasets used in evaluation

Datasets	Organ	Cells	Genes	Class	Platform	Reference
Adam	Kidney	3660	23797	8	Drop-seq	Adam et al.[1]
Bach	Gammary Gland	23184	19965	8	10x	Bach et al.[2]
Heart LV	Heart	107249	33538	12	10x	Kanemaru et al.[3]
Klein	Embryonic Stem Cell	2717	24047	4	inDrop	Klein et al.[4]
Macosko	Retina	44808	24658	12	Drop-Seq	Macosko et al.[5]
Muraro	Pancreas	2122	19046	9	CEL-seq2	Muraro et al.[6]
Plasschaert	Trachea	6977	28205	8	inDrop	Plasschaert et al.[7]
Pollen	Tissues	301	21721	11	SMARTer	Pollen et al.[8]
Quake 10x Bladder	Bladder	2500	23341	4	10x	Schaum et al.[9]
Quake 10x Limb Muscle	Limb Muscle	3909	23341	6	10x	Schaum et al.[9]
Quake 10x Spleen	Spleen	9522	23341	5	10x	Schaum et al.[9]
Quake Smart-seq2 Diaphragm	Diaphragm	870	23341	5	Smart-seq2	Schaum et al.[9]
Quake Smart-seq2 Heart	Heart	4365	23341	8	Smart-seq2	Schaum et al.[9]
Quake Smart-seq2 Limb Muscle	Limb Muscle	1090	23341	6	Smart-seq2	Schaum et al.[9]
Quake Smart-seq2 Trachea	Trachea	1350	23341	2	Smart-seq2	Schaum et al.[9]
Romanov	Hypothalamus	2881	21143	7	SMARTer	Romanov et al.[10]
Tosches turtle	Brain	18664	23500	15	Drop-seq	Tosches et al.[11]
Young	Kidney	5685	33658	11	10x	Young et al.[12]

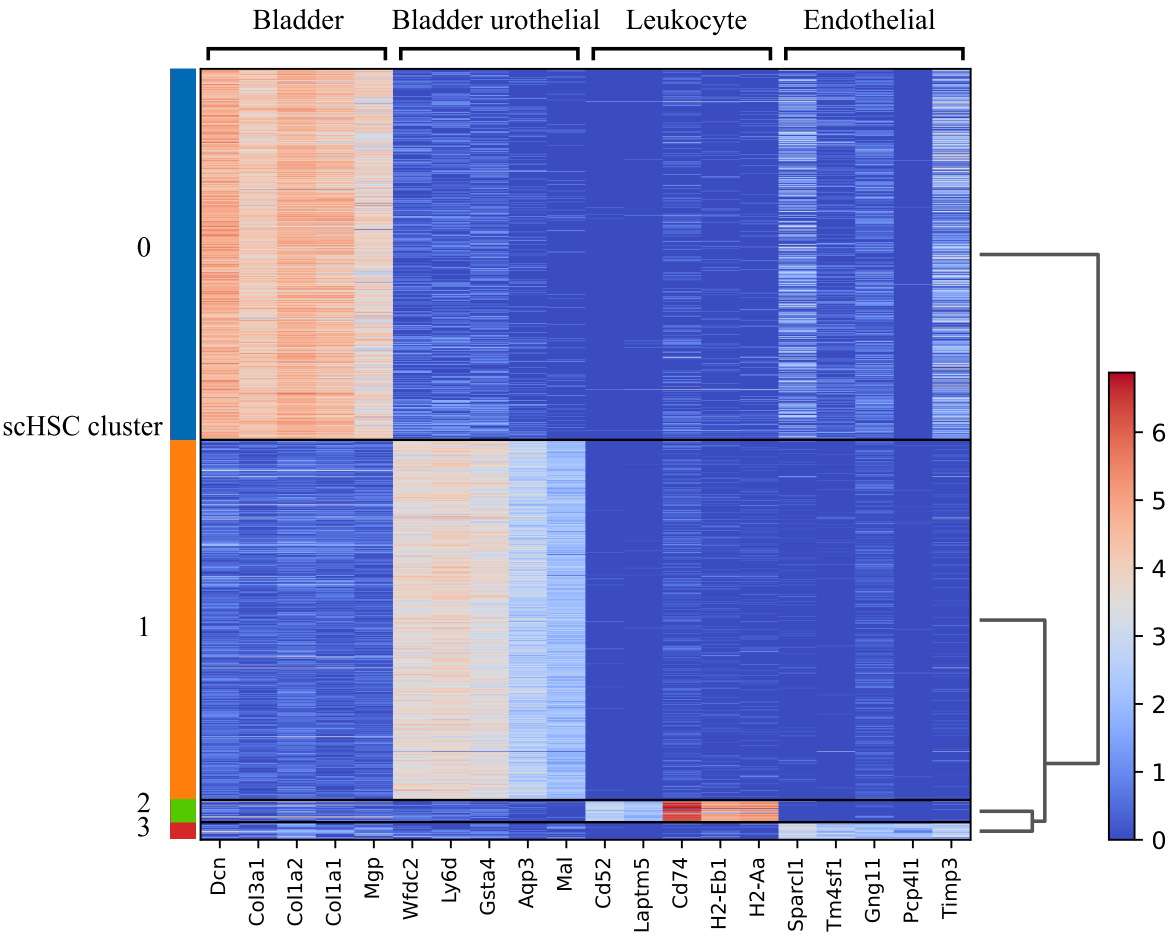

Figure S3: Heatmap of marker gene expression across clusters identified by scHSC on the Quake 10x Bladder dataset. Each column corresponds to a marker gene identified by differential expression analysis using *sc.tl.rank_genes_groups*. The distinct expression patterns highlight the biological specificity of the scHSC-derived clusters.

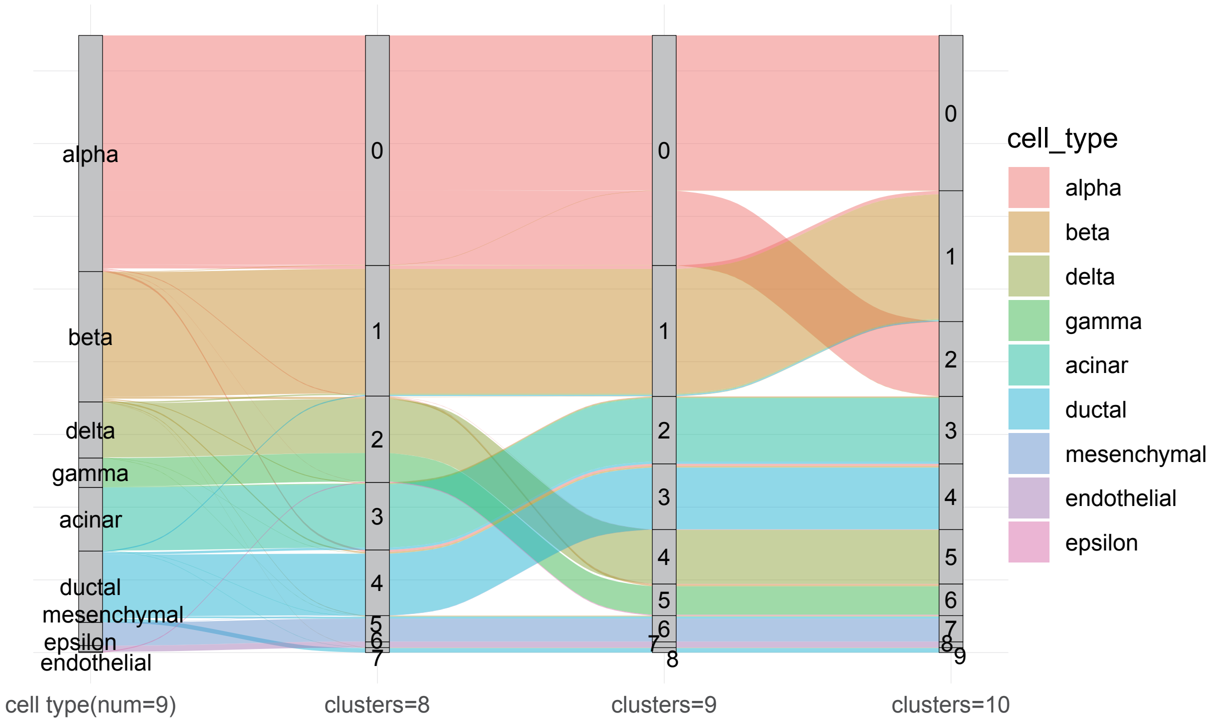

Figure S4: Sankey plot illustrating the robustness of scHSC to cluster number mis-specification on the Muraro dataset. The ground-truth labels (9 cell types) are compared with clustering results from scHSC using 8, 9, and 10 clusters. When using 8 clusters, delta and gamma cells are merged due to their biological similarity. With 10 clusters, alpha cells are slightly over-partitioned. Most cell identities remain consistent across settings, highlighting the model's robustness to cluster number perturbation.

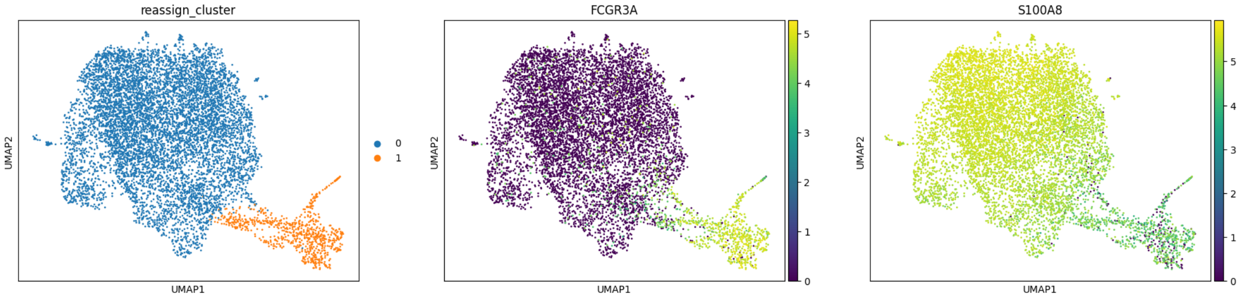

Figure R1: scHSC could distinguished FCGR3A-positive and S100A8-positive cells, which correspond to known markers of non-classical and classical monocytes, respectively. Moreover, a potential transition pattern between the two populations was observed, consistent with prior biological knowledge.

Supplementary Table S2: Evaluation of initialization strategies and PCA-based pseudo-label initialization on the Muraro dataset. Models were trained using two initialization methods (kaiming uniform and kaiming normal) with 10 different random seeds each. Performance metrics (ARI, NMI, and ACC) are reported as means with standard deviations in parentheses. Comparisons are made between models with and without PCA-based pseudo-label initialization during the first 10 training iterations.

NMI	Network initialization / PCA embedding	Kaiming normal	Kaiming uniform	Average
	early_stage	0.883598387	0.881747422	0.882673
	never	0.883670704	0.884325369	0.883998
ARI	Network initialization / PCA embedding	Kaiming normal	Kaiming uniform	Average
	early_stage	0.922882305	0.921466452	0.922174379
	never	0.923132276	0.9231316	0.923131938
ACC	Network initialization / PCA embedding	Kaiming normal	Kaiming uniform	Average
	early_stage	0.952780396	0.95131951	0.952049953
	never	0.952874647	0.954948162	0.953911404

Supplementary Table S3: Clustering performance comparison of scHSC under fixed weighting parameters (ω = 1, 5, 10) and the default dynamic weighting strategy across four datasets (Adam, Muraro, Pollen, and Quake Smart-seq2 Diaphragm). Each setting was run 10 times with different random seeds; values in parentheses indicate standard deviations. The best results for each dataset are highlighted in bold, and the second-best are underlined.

Dataset	ZINB loss	Weight	NMI	ARI	ACC
Adam	~0.6	Adaptive	**0.8688(0.0164)**	**0.8673(0.0288)**	**0.9371(0.0134)**
		1	0.8589(0.0133)	0.852(0.0254)	0.9284(0.0146)
		5	0.8551(0.0124)	0.846(0.0231)	0.9254(0.0135)
		10	<u>0.867(0.014)</u>	<u>0.866(0.0238)</u>	<u>0.9362(0.012)</u>
Muraro	~1.4	Adaptive	<u>0.8756(0.0224)</u>	<u>0.909(0.0353)</u>	<u>0.9383(0.0398)</u>
		1	**0.8761(0.0221)**	**0.9097(0.0349)**	**0.9385(0.0397)**
		5	0.8707(0.0259)	0.8904(0.066)	0.9272(0.05)
		10	0.8708(0.0253)	0.8905(0.0658)	0.9272(0.0502)
Qs Diaphragm	~12	Adaptive	**0.9365(0.0237)**	**0.9667(0.0243)**	**0.9761(0.0155)**
		1	<u>0.9204(0.0268)</u>	<u>0.9534(0.0318)</u>	<u>0.9619(0.0305)</u>
		5	0.916(0.0306)	0.9519(0.0356)	0.9574(0.0324)
		10	0.9166(0.0302)	0.9522(0.0346)	0.9575(0.0321)
Pollen	~24	Adaptive	**0.9233(0.0083)**	**0.849(0.0203)**	**0.8596(0.0281)**
		1	<u>0.919(0.0115)</u>	<u>0.8325(0.0301)</u>	<u>0.8423(0.0396)</u>
		5	0.9178(0.0116)	0.8317(0.0306)	0.8396(0.0414)
		10	0.9186(0.0122)	0.837(0.0305)	0.8454(0.0423)

Supplementary Table S4: Clustering evaluation metrics (Compactness and Separation) calculated on standardized embeddings across multiple datasets and methods. Lower Compactness values indicate tighter intra-cluster cohesion, while higher Separation values reflect better inter-cluster separation. Best performances are highlighted in bold, and worst performances are underlined**.**

Dataset	DCA	DESC	PCA+ louvain	scDeep cluster	scGAE	scHSC	scMAE	scTAG	sczi Desk
Adam	**0.098**	7.145	<u>44.176</u>	**0.66**	7.125	12.726	<u>61.716</u>	**0.138**	<u>17.814</u>
Bach	**0.028**	1.321	<u>44.163</u>	**0.518**	0	11.774	<u>64.952</u>	**0**	<u>17.376</u>
Klein	**0.081**	4.15	<u>44.341</u>	**0.862**	4.027	11.82	<u>27.263</u>	**0.069**	<u>4.101</u>
Macosko	**0.156**	15.14	<u>47.588</u>	**2.326**	6.188	14.072	<u>84.992</u>	**0.208**	<u>22.65</u>
Muraro	**0.128**	2.66	<u>42.061</u>	**0**	0	20.372	<u>76.268</u>	**0**	<u>31.999</u>
Plasschaert	**0.035**	7.626	<u>42.928</u>	**0.736**	3.975	9.217	<u>24.863</u>	**0.141**	<u>10.94</u>
Pollen	**0.041**	3.223	<u>44.568</u>	**0.47**	7.003	18.697	<u>47.89</u>	**0.246**	<u>19.429</u>
Qx Bladder	**0.016**	5.553	<u>41.538</u>	**0.344**	2.204	4.448	<u>22.72</u>	**0.002**	<u>7.599</u>
Qx Muscle	**0.143**	19.032	<u>47.521</u>	**6.969**	9.808	15.235	<u>58.675</u>	**0.098**	<u>18.675</u>
Qx Spleen	**0.044**	15.396	<u>45.275</u>	**0.77**	5.324	15.038	<u>70.334</u>	**0.05**	<u>18.935</u>
Qs Diaphragm	**0.219**	1.798	<u>46.729</u>	**1.02**	11.96	21.301	<u>75.91</u>	**0.344**	<u>20.163</u>
Qs Heart	**0.079**	13.564	<u>46.163</u>	**3.285**	8.408	11.883	<u>39.54</u>	**0.056**	<u>14.188</u>
Qs Muscle	**0.031**	11.919	<u>43.612</u>	**0.729**	7.415	14.182	<u>57.599</u>	**0.083**	<u>18.294</u>
Qs Trachea	**0.049**	10.971	<u>45.807</u>	**2.13**	6.464	11.846	<u>38.654</u>	**0.02**	<u>11.114</u>
Romanov	**0.211**	23.208	<u>47.115</u>	**2.142**	10.535	24.576	<u>83.439</u>	**0.08**	<u>21.487</u>
Tosches turtle	**0.114**	12.248	<u>45.463</u>	**1.873**	8.101	13.489	<u>52.295</u>	**0.224**	<u>17.327</u>
Young	**0.015**	0.426	<u>39.005</u>	**0.217**	0	10.206	<u>47.07</u>	**0**	<u>16.706</u>
heart_lv	**0.061**	3.684	<u>41.944</u>	**0.455**	6.73	13.691	<u>59.244</u>	**0.097**	<u>18.842</u>

Supplementary Table S5: Clustering evaluation metrics (Compactness and Separation) calculated on standardized embeddings across multiple datasets and methods. Lower Compactness values indicate tighter intra-cluster cohesion, while higher Separation values reflect better inter-cluster separation. Best performances are highlighted in bold, and worst performances are underlined.

Dataset	DCA	DESC	PCA+ louvain	scDeep cluster	scGAE	scHSC	scMAE	scTAG	sczi Desk
Adam	14.267	255.042	52.932	**317.007**	63.928	154.852	**673.755**	133.644	133.812
Bach	12.1	270.107	106.053	**367.917**	0	167.211	**1217.546**	0	195.728
Klein	7.542	87.846	14.107	**159.323**	33.521	73.29	**191.645**	72.488	40.815
Macosko	27.779	495.942	1378.176	**0**	0	188.7	**7690.414**	0	20.116
Muraro	13.26	357.453	165.712	**528.521**	125.605	226.238	**2568.626**	344.605	619.614
Plasschaert	14.789	271.449	298.529	**454.314**	68.51	145.202	**2729.49**	451.158	553.207
Pollen	18.992	287.741	176.033	**358.055**	167.954	294.47	**1521.414**	164.507	302.962
Qx Bladder	6.501	56.105	14.043	**163.306**	21.644	75.933	**761.635**	170.181	271.158
Qx Muscle	10.205	99.492	38.688	**227.254**	60.579	124.082	**426.146**	113.474	106.396
Qx Spleen	19.304	260.21	45.688	**277.372**	15.168	87.225	**1133.644**	171.796	539.547
Qs Diaphragm	10.996	152.453	43.755	**169.058**	40.805	120.354	**860.186**	162.3	179.716
Qs Heart	14.614	388.449	290.532	**230.093**	69.349	164.082	**2489.717**	282.226	296.519
Qs Muscle	10.05	194.858	44.577	**198.944**	56.859	154.733	**998.228**	180.623	234.143
Qs Trachea	8.374	89.779	26.963	**245.814**	17.636	36.833	**613.003**	102.035	152.161
Romanov	11.308	229.384	60.826	**274.771**	57.095	178.361	**1146.267**	246.955	223.598
Tosches turtle	27.123	575.065	1075.737	**869.618**	0	330.845	**4862.448**	0	465.181
Young	19.819	410.241	226.197	**405.534**	97.829	214.675	**1071.541**	533.876	281.903
heart_lv	17.463	410.977	1052.317	**0**	0	270.753	**4397.49**	0	5248.484

References

[1] ADAM M, POTTER A S, POTTER S S. Psychrophilic proteases dramatically reduce single-cell RNA-seq artifacts: a molecular atlas of kidney development[J]. Development, 2017, 144(19): 3625-3632.

[2] BACH K, PENSA S, GRZELAK M, et al. Differentiation dynamics of mammary epithelial cells revealed by single-cell RNA sequencing[J]. Nature communications, 2017, 8(1): 1-11.

[3] KANEMARU K, CRANLEY J, MURARO D, et al. Spatially resolved multiomics of human cardiac niches[J]. Nature, 2023.

[4] KLEIN A M, MAZUTIS L, AKARTUNA I, et al. Droplet barcoding for single-cell transcriptomics applied to embryonic stem cells[J]. Cell, 2015, 161(5): 1187-1201.

[5] MACOSKO E Z, BASU A, SATIJA R, et al. Highly parallel genome-wide expression profiling of individual cells using nanoliter droplets[J]. Cell, 2015, 161(5): 1202-1214.

[6] MURARO M J, DHARMADHIKARI G, GRÜN D, et al. A single-cell transcriptome atlas of the human pancreas[J]. Cell systems, 2016, 3(4): 385-394.

[7] PLASSCHAERT L W, ŽILIONIS R, CHOO-WING R, et al. A single-cell atlas of the airway epithelium reveals the CFTR-rich pulmonary ionocyte[J]. Nature, 2018, 560(7718): 377-381.

[8] POLLEN A A, NOWAKOWSKI T J, SHUGA J, et al. Low-coverage single-cell mRNA sequencing reveals cellular heterogeneity and activated signaling pathways in developing cerebral cortex[J]. Nature biotechnology, 2014, 32(10): 1053-1058.

[9] SCHAUM N, KARKANIAS J, NEFF N F, et al. Single-cell transcriptomics of 20 mouse organs creates a Tabula Muris: The Tabula Muris Consortium[J]. Nature, 2018, 562(7727): 367.

[10] ROMANOV R A, ZEISEL A, BAKKER J, et al. Molecular interrogation of hypothalamic organization reveals distinct dopamine neuronal subtypes[J]. Nature neuroscience, 2017, 20(2): 176-188.

[11] TOSCHES M A, YAMAWAKI T M, NAUMANN R K, et al. Evolution of pallium, hippocampus, and cortical cell types revealed by single-cell transcriptomics in reptiles[J]. Science, 2018, 360(6391): 881-888.

[12] YOUNG M D, MITCHELL T J, VIEIRA BRAGA F A, et al. Single-cell transcriptomes from human kidneys reveal the cellular identity of renal tumors[J]. science, 2018, 361(6402): 594-599.
